# Speed-Dependent Modulation of the Locomotor Behavior in Adult Mice Reveals Attractor and Transitional Gaits

**DOI:** 10.3389/fnins.2016.00042

**Published:** 2016-02-23

**Authors:** Maxime Lemieux, Nicolas Josset, Marie Roussel, Sébastien Couraud, Frédéric Bretzner

**Affiliations:** ^1^Centre de Recherche du CHU de Québec, CHUL-NeurosciencesQuébec, QC, Canada; ^2^Department of Psychiatry and Neurosciences, Faculty of Medicine, Université LavalQuébec, QC, Canada

**Keywords:** kinematic, steady-state, mouse, speed, locomotor gaits, graph analysis

## Abstract

Locomotion results from an interplay between biomechanical constraints of the muscles attached to the skeleton and the neuronal circuits controlling and coordinating muscle activities. Quadrupeds exhibit a wide range of locomotor gaits. Given our advances in the genetic identification of spinal and supraspinal circuits important to locomotion in the mouse, it is now important to get a better understanding of the full repertoire of gaits in the freely walking mouse. To assess this range, young adult C57BL/6J mice were trained to walk and run on a treadmill at different locomotor speeds. Instead of using the classical paradigm defining gaits according to their footfall pattern, we combined the inter-limb coupling and the duty cycle of the stance phase, thus identifying several types of gaits: lateral walk, trot, out-of-phase walk, rotary gallop, transverse gallop, hop, half-bound, and full-bound. Out-of-phase walk, trot, and full-bound were robust and appeared to function as attractor gaits (i.e., a state to which the network flows and stabilizes) at low, intermediate, and high speeds respectively. In contrast, lateral walk, hop, transverse gallop, rotary gallop, and half-bound were more transient and therefore considered transitional gaits (i.e., a labile state of the network from which it flows to the attractor state). Surprisingly, lateral walk was less frequently observed. Using graph analysis, we demonstrated that transitions between gaits were predictable, not random. In summary, the wild-type mouse exhibits a wider repertoire of locomotor gaits than expected. Future locomotor studies should benefit from this paradigm in assessing transgenic mice or wild-type mice with neurotraumatic injury or neurodegenerative disease affecting gait.

## Introduction

Locomotion results from an interplay between biomechanical constraints of the muscles attached to the axial and appendicular skeleton and the neuronal circuit that controls these muscles. Over the last decade, advances in mouse genetics have allowed us to identify the spinal interneuronal circuits controlling muscles underlying motor and locomotor functions. Neonatal *in vitro* and adult *in vivo* locomotor studies using genetic manipulations (e.g., signaling cues involved in neural circuit formation or ablations of genetically identified neuronal populations) have revealed important information about the neural control of locomotion, especially the left-right alternation of the hindlimbs (Kullander et al., 2001a,b; Kullander, 2003; Lanuza et al., 2004; Crone et al., 2008; Zhang et al., 2008; Rabe et al., 2009; Andersson et al., 2012; Bernhardt et al., 2012; Talpalar et al., 2013; Borgius et al., 2014). However, less is known about the forelimbs and even less about locomotor gaits.

Historically, locomotor gaits were identified as symmetrical vs. asymmetrical according to their footfall pattern (Hildebrand, 1976). A gait was defined as symmetrical when it could be described by only half the step cycle, the other half being symmetrical to the first half. Conversely, asymmetrical gaits could not be described by half the cycle. Using this paradigm, it has been shown that most quadrupeds, such as monkeys, horses, dogs, cats, and rats, display a large repertoire of locomotor gaits from walk, to pace, to trot, to gallop (Cohen and Gans, 1975; Grillner, 1975; Miller et al., 1975; Hildebrand, 1976; Dunbar, 2004; Abourachid et al., 2007; Maes and Abourachid, 2013). The full range of the locomotor repertoire of the mouse has not yet been established. Nevertheless, these different gaits, displaying distinct locomotor rhythms and patterns, are likely generated by the same neuronal circuit across the vertebrate phylogeny (Orlovsky et al., 1999).

Previously, *in vivo* locomotor studies have shown that if some mutant mice can synchronize their hindlimb (i.e., hop, gallop, or bound) at various speeds, their wild-type littermates systematically alternate their hindlimb (i.e., walk or trot) at locomotor speeds up to 8 Hz and above (Talpalar et al., 2013; Borgius et al., 2014). Although gallop and bound occur in wild-type mice during brief acceleration phases on a treadmill (Herbin et al., 2004, 2006, 2007), on a catwalk (Bellardita and Kiehn, 2015), and on a catwalk following noxious stimulations (Serradj and Jamon, 2009), these gaits only occur over a few strides, thus raising some concerns as to whether mice can sustain galloping and bounding. Since most quadruped mammals can sustain galloping at high speed, we therefore hypothesized that wild-type mice should be able to maintain galloping and bounding at high speed.

Our experimental approach has been to assess locomotor gaits in young adult C57BL/6J mice during treadmill locomotion over a wide range of speeds. The advantage of treadmill locomotion over catwalk over-ground locomotion is that by controlling the speed it allows one to analyse slight accelerations or decelerations of the mouse while walking or running at a steady speed. To identify and objectively characterize locomotor gaits, we combined the inter-limb coupling and the duty cycle of the stance phase of individual steps according to the treadmill speed. Assuming that locomotion is a dynamic process, we hypothesized that certain locomotor gaits, by their occurrence, their robustness, and their stability, should emerge as preferential gaits (i.e., attractor gaits), while others would occur as transitional gaits (e.g., during transitions from walking to running or during initiation of locomotion).

Here we show that wild-type mice can sustain gallops and bounds at high running speed. Moreover, we identified attractor gaits occurring over a wide range of speeds and transitional gaits over a narrower range of speeds. Using graph analysis, a mathematical approach to describing the elements and interactions within a complex network (Strogatz, 2001; Bullmore and Sporns, 2009), we demonstrated that transitions between gaits are not random, but predictable. Using this new paradigm to better identify and characterize locomotor gaits, our study should help future locomotor studies of transgenic mice or wild-type mice impaired by neurotraumatic injury or neurodegenerative disease.

## Materials and methods

Six adult C57BL/6J mice (>3 weeks old) of either sex were used in this study. All procedures were performed according to the guidelines of the Canadian Council on Animal Care and were approved by the local committee of Université Laval (CPAUL and CPAC).

### Kinematic recording

Mice were trained to walk on a commercially available single-lane mouse treadmill (LE 8700 Series, Panlab). The inner dimensions of the lane were 32 × 5 cm. Speed could be adjusted from 5 to 150 cm/s. The electrified grid at the rear of the lane was set at the minimal intensity (0.1 mA) to motivate locomotion of mice on the belt. First, mice were allowed to acclimate quietly on the lane for 20–30 min. They were then introduced to walk at 10–15 cm/s for 5 min. At that stage, the mice kept walking on the treadmill belt to avoid the electrified grid. Among the group of nine mice used during the training phase, six learned to avoid the electrified grid. The three remaining mice were excluded from the study. Mice were walked at increasing speed. Once they reached 100 cm/s, they were tested 3 times at each speed to obtain at least 10 contiguous strides (bouts of 10–60 s depending on the speed). All mice were filmed on the left and right sides by high-frequency (200 frames/s) cameras (Genie HM640, Dalsa Teledyne) during treadmill locomotion. To study inter-limb coordination over a wide range of locomotor speeds, mice were tested at treadmill belt speeds of 5, 10, 15, 30, 45, 60, 75, 90, 105, 120, 135, and 150 cm/s. To investigate the limb trajectory, mice walked at low (15 cm/s), intermediate (45 cm/s), and high speeds (90 cm/s) with reflective markers. Under isoflurane anesthesia (2–3%), limbs were shaved and reflective markers (2 mm diameter) were glued on the iliac crest, hip, knee, ankle, and metatarsophalangeal (MTP) joints and toe for the hindlimb, and on the scapula, shoulder, elbow, and metacarpophalangeal joints and toe for the forelimb. Films were digitized with StreamPix 6.0 (Norpix) and analyzed offline.

### Kinematic analysis

For our kinematic studies, videos were analyzed by using custom-designed software (graciously provided by Drs. S. Rossignol and T. Drew, Université de Montréal) during steady-state treadmill locomotion, thus avoiding the acceleration and deceleration phases observed with a catwalk setup. The timing of foot lifts and contacts for all four limbs, as well as the two-dimensional spatial coordinates of joints, were manually extracted at a resolution of 5 ms (200 samples/s). Temporal and spatial data were exported and processed with custom-written routines in Matlab (MathWorks). We first evaluated basic locomotor parameters. The step cycle was defined by two successive foot contacts from the reference limb (here, the left hindlimb) to determine the instantaneous step frequency. The step cycle was divided in two phases: the stance phase initiated when the foot of a limb made contact with the ground, thus supporting a part of the body weight, and terminated when the foot was lifted at the onset of the swing phase. The duty cycle of the stance phase was computed as the stance duration divided by the cycle duration and expressed as a percentage. The phase value corresponded to the time of foot contact (HL coupling in **Figure 2A**, out-of-phase walk gait diagram) relative to the reference limb step cycle. Phase values range from 0 to 1. Phase values of 0 or 1 indicate a perfect in-phase coupling (i.e., synchrony), while a phase value of 0.5 indicates a perfect anti-phase coupling (i.e., strict alternation).

**Figure 1:**
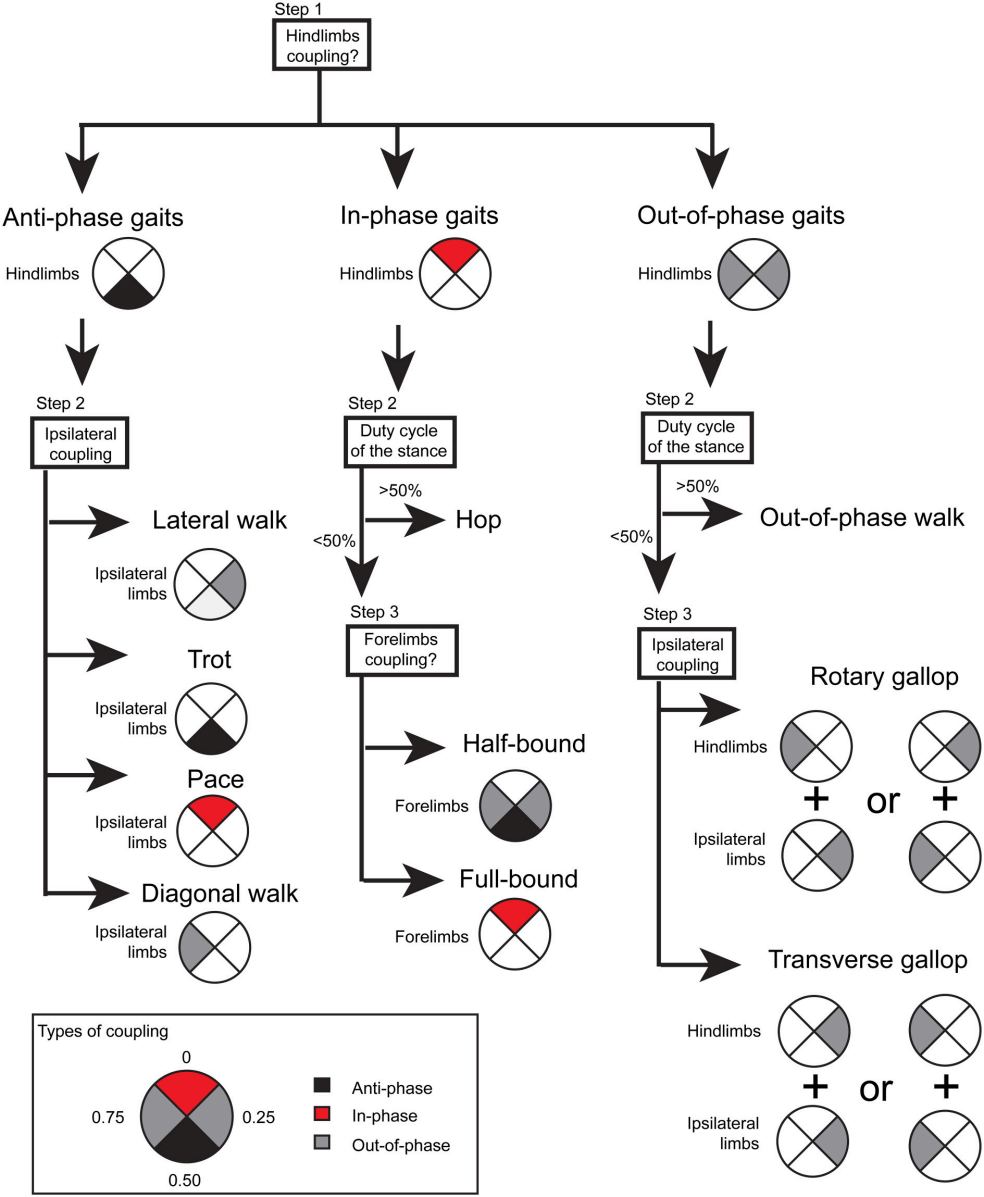
**Gait identification**. The procedure depicts the architecture of the automated routine identifying the gait. Step 1 is based on the hindlimbs coupling (left side as reference). Gait may be anti-phase (black), in-phase (red), or out-of-phase (gray). If the step cycle corresponded to a hindlimb anti-phase gait, step 2 used the ipsilateral limbs coupling (hindlimb as reference) to identify the gait: lateral walk (low out-of-phase coupling), trot (anti-phase), pace (in-phase), or diagonal walk (high out-of-phase coupling). If the step cycle corresponded to a hindlimbs anti-phase or in-phase coupling, step 2 differentiated walking (duty cycle of the stance <50%) from running gaits (duty cycle of the stance >50%). For running gaits, a third step was required to differentiate half-bound from full-bound and rotary gallop from transverse gallop. The box presents the definition for the types of coupling.

Based on previous studies comparing several quadruped species (Hildebrand, 1968, 1977; Heglund and Taylor, 1988; Abourachid et al., 2007) or focusing on dogs (Maes and Abourachid, 2013) or mice (Herbin et al., 2004), we identified and classified eight gaits: lateral walk, trot, rotary gallop, transverse gallop, half-bound, full-bound, hop, and out-of-phase walk (see procedure in Figure 1). This last gait has not been previously described. To assign a step cycle in a particular gait, we used as criteria the phase values of homologous limbs and ipsilateral limbs and the duty cycle of the hindlimb stance (Table 1). Once all step cycles were identified, we computed the mean phase and vector length (r) of hind-, fore-, ipsi-, and diagonal couplings of each gait. Coupling was identified as in-phase (phase = 0 ± 0.125), anti-phase (0.5 ± 0.125) or out-of-phase (low coupling: 0.125–0.375, high coupling: 0.625–0.875). We chose ± 0.125 (or 45°) to equally distribute coupling values among quadrants. For the intra-limb coordination, we analyzed the spatial and temporal data of reflective markers placed on fore- and hindlimb joints of 6 mice at 3 treadmill speeds (15, 45, and 90 cm/s). We calculated the stride length and height of fore- and hindfoot, as well as the maximal speed and acceleration of the limb trajectory. The product of the speed of the treadmill belt and the duration of the swing phase were added to the apparent stride length to get the real stride length.

**Table 1:** **Basic locomotor parameters for each gait**.

	**OPW-lo** **OPW-hi**	**Hop-lo** **Hop-hi**	**LW**	**T**	**RG** **TG**	**HB** **FB**
Step frequency (Hz)	1.6 ± 0.8 8.2 ± 1.0	1.8 ± 1.4 10.1 ± 2.5	2.6 ± 0.8	4.8 ± 2.0	8.5 ± 1.1 9.3 ± 1.6	9.6 ± 1.2 10.1 ± 1.5
Stance duration (ms)	664 ± 440 62 ± 21	1040 ± 958 70 ± 12	326 ± 180	176 ± 156	49 ± 11 48 ± 13	40 ± 7 35 ± 6
Swing duration (ms)	128 ± 41 54 ± 10	88 ± 36 43 ± 10	121 ± 58	95 ± 32	71 ± 12 63 ± 10	66 ± 13 66 ± 15
Duty cycle of the stance phase (%)	80.6 ± 10.7 56.2 ± 6.0	86.8 ± 7.5 58.3 ± 6.8	72.3 ± 12.1	60.1 ± 15.6	40.6 ± 5.9 42.7 ± 6.3	38.0 ± 6.7 34.9 ± 6.5

*Data are presented as mean ± SD. Gaits: OPW-lo, out-of-phase walk at low step frequency; OPW-hi, out-of-phase walk at high step frequency; Hop-lo, hop at low step frequency; Hop-hi, hop at high step frequency; LW, lateral walk; T, trot; RG, rotary gallop; TG, transverse gallop; HB, half-bound; FB, full-bound*.

### Graph analysis

Graph analysis is a technique often applied to the study of complex network (Strogatz, 2001; Mason and Verwoerd, 2007; Bullmore and Sporns, 2009; Ma'Ayan, 2009). Networks are represented as nodes (or vertices) connected by links (or edges). Gaits were defined as nodes, and transitions between gaits as edges. Graphs were constructed at each speed. The weight of a transition from one node to another (e.g., from node A to node B) was calculated as the ratio of this path occurrence on all transitions from the node of origin (node A). In the context of our study, we investigated for all speeds: (1) the probability that a gait remains the same from cycle to cycle (stability), (2) the probability that other gaits converge toward a specific gait (attractiveness), and (3) the probability that when a mouse breaks away from a given gait, it tends to move toward another gait (predictability of transition). For all speeds, we calculated the probability of stability of a gait as the ratio of consecutive step cycles corresponding to the same gait on the total number of step cycles. The attractiveness of a gait corresponded to probability that a step cycle of any other gait changed to this gait. The predictability of transition was computed as the probability of observing a transition from one gait to another. Somewhat similar to the measure of attractiveness, the predictability also included the probability of transition between gaits separated by 2-4 edges (the probability was then calculated as the product of all edges).

### Statistics

Circular statistics were used to evaluate the phase values of forelimbs, hindlimbs, homolateral left limbs, and diagonal limbs (opposite left hindlimb and right forelimb) (Drew and Doucet, 1991; Kiehn and Kjaerulff, 1996; Zar, 1996). The significance of step frequencies, stride length, and height was evaluated with Kruskal-Wallis (due to unequal variance of data as evaluated by the Bartlett test) with *post-hoc* paired comparison with the Tukey's Honestly Significant Difference (HSD) test.

## Results

### Locomotor gaits: A revised paradigm

**Figure 2:**
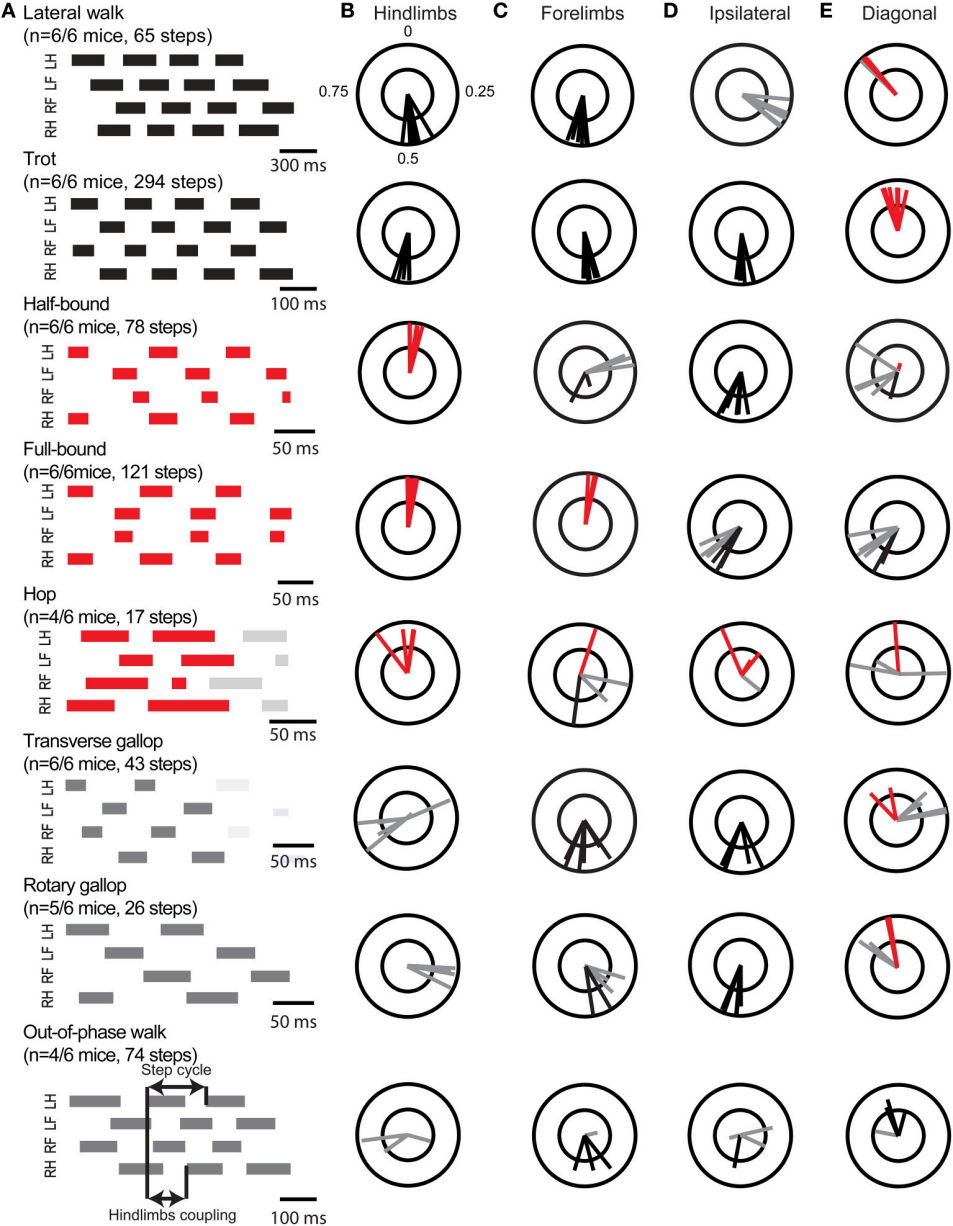
**Gait identification and interlimb coordination. (A)** Gait diagrams of locomotor patterns identified in mouse locomotion. Stance phases are represented by thick lines, swing phases correspond to the gaps between them. Gait diagrams and polar plots are color-coded according to the interlimb coupling (anti-phase in black, in-phase in red, and out-of-phase in gray). Limbs are in rows, from top to bottom: the left hindlimb (LH), left forelimb (LF), right forelimb (RF), and right hindlimb (RH). As shown during the out-of-phase walk, phase coupling is the ratio between the lag (i.e., time between a limb contact and its opposite limb contact on the belt) and the step cycle duration. In this case, there is a lag of the right side in relation to the left side. Polar plots in (**B–E)** show the mean phase coupling of all mice for each gait for **(B)** the left-right hindlimbs (left hindlimb as reference), **(C)** the left-right forelimbs (left forelimb as reference), **(D)** the left forelimb–left hindlimb (ipsilateral, left hindlimb as reference) and **(E)** right forelimb–left hindlimb (diagonal, left hindlimb as reference). Each vector indicates the mean phase (direction) and robustness (radius) of the coupling. The color of the vector indicates whether the mean coupling is in-phase (red), anti-phase (black), or out-of-phase (gray).

Figure 2A shows typical examples of different gaits in a mouse with left hindlimb, left forelimb, right forelimb, and right hindlimb contacts representing the stance phase, while gaps represent the swing phase. The locomotion of six mice was assessed during steady-state locomotion (at least 10 contiguous steps) at treadmill speeds ranging from 5 to 150 cm/s. Because gaits might change from cycle to cycle, we analyzed the locomotor gait based on individual steps using the phase of the interlimb coupling and the duty cycle of the stance phase. Instead of using a definition based on the symmetry/asymmetry of the footfall of all four limbs across the step cycle (Hildebrand, 1976), we opted to use terms referring directly to the phase of the interlimb coupling. Based on the type of coupling between hindlimbs, forelimbs, and ipsilateral limbs, we identified 8 gaits: 2 gaits with an anti-phase hindlimb coupling, shown in black; 3 gaits with an in-phase hindlimb coupling, shown in red; and 3 gaits with an out-of-phase hindlimb coupling, shown in gray (Figure 2B).

#### Gaits with an anti-phase hindlimb coupling

As shown by the polar plots adjacent to their gait diagrams (Figures 2B–E), anti-phase gaits, such as the lateral walk and the trot, were identified by a robust anti-phase coupling of their left-right hindlimbs and forelimbs. The fore-hindlimb coupling was out-of-phase during lateral walk (phase < 0.5, Supplementary Video 1), while it was in anti-phase during trot (Figure 1D, Supplementary Video 2). The two other anti-phase gaits, the pace and the diagonal walk (Hildebrand, 1976; Abourachid et al., 2007), were never observed in C57BL/6J mice.

#### Gaits with an in-phase hindlimb coupling

These gaits corresponded to half-bound, full-bound, and hop (Grillner, 1975; Hildebrand, 1976). The full-bound was distinguished from the half-bound by a robust in-phase coupling of the left-right forelimbs (Supplementary Videos 3, 4). The duty cycle of the stance phase was inferior to 50%, which was indicative of running gaits (Figure 1; see also Figures 4, 8 from Hildebrand, 1976). The hop was observed in 4 out of 6 mice and was characterized by a looser in-phase hindlimbs coupling. The couplings of fore-, ipsilateral, or diagonal limbs was quite variable from mouse to mouse, thus identifying it as a distinct gait from half-bound and full-bound but also as a loosely organized gait (Supplementary Video 5).

#### Gaits with an out-of-phase hindlimb coupling

Based on the duty cycle, we were able to identify and characterize two more running gaits, the transverse and the rotary gallop, for which the hindlimb coupling was out-of-phase (Supplementary Video 6). While the out-of-phase coupling of hindlimbs was more variable in the transverse gallop than in rotary gallop, the anti-phase coupling of forelimbs was more robust. In addition, we also found another gait with an out-of-phase coupling of hindlimbs but with a duty cycle of the stance phase superior to 50% (walking gait). The direction and robustness of coupling between limbs was variable across mice, thus suggesting a less stable coordination of left-right activities at the cervical level and between cervical and lumbar half-centers. To distinguish it from lateral walk, we named this gait “out-of-phase walk” (Supplementary Video 7).

### Attractor vs. transitional gaits

**Figure 3:**
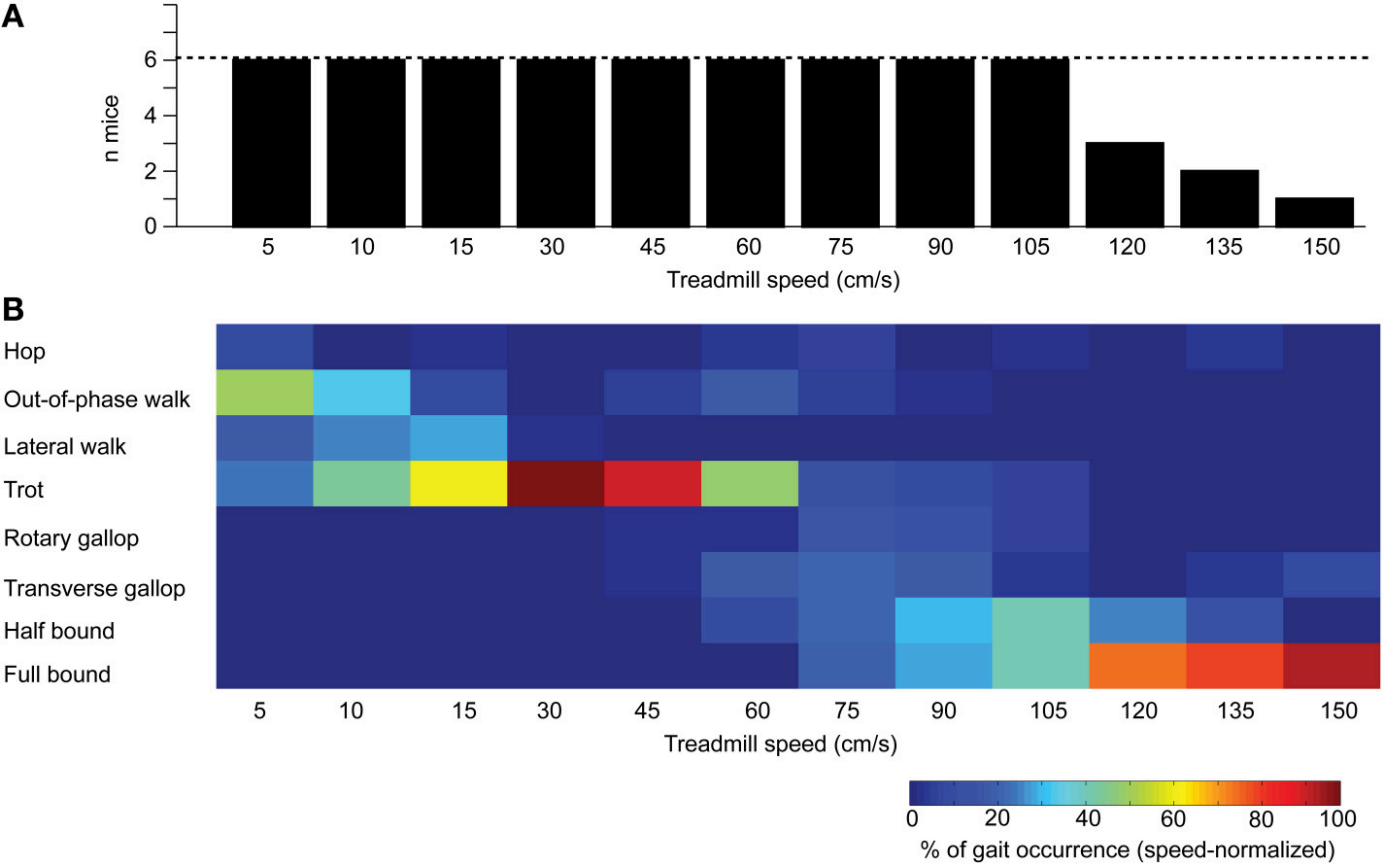
**Occurrence of gaits at different treadmill speeds. (A)** Bar graph illustrating the number of mice walking or running for more than 10 consecutive steps at a given speed. Dashed line indicates the number of tested mice (*n* = 6) **(B)** Color-coded matrix of the percentage of occurrence of a gait (row) at each speed (column). The sum of a column equals 100%.

We next hypothesized that, given their high occurrence, preferential gaits could be considered as attractor gaits and should occur over a wide range of speeds, whereas the others would emerge as transitional gaits, occurring less often and over a narrower range of speeds. All mice could run up to 105 cm/s, and the number of mice running decreased beyond that speed (Figure 3A). As illustrated by the color-coded matrix in Figure 3B, two attractor gaits emerged: trot at walking speed (30 cm/s) and full-bound at running speed (>120 cm/s). The out-of-phase walk was the dominant gait at speeds below 15 cm/s, but never at the extent observed for trot and full-bound. A somewhat similar phenomenon occurred at high speeds with half-bound. Although never dominant over the full-bound, half-bound occurred in similar proportion to full-bound at 90 and 105 cm/s. Mice running at 120–150 cm/s had a clear preference for full-bound. Although we cannot exclude the possibility that full-bound might have been over-represented at the expense of half-bound due to the decreasing number of mice running at and beyond 120 cm/s (Figure 3A), these results suggest that full-bound is a prerequisite to achieving a greater velocity. Overall, these results highlight the existence of two attractor gaits: trot and full-bound.

The other gaits barely exceeded an occurrence of 30% at any speed. Surprisingly, lateral walk was found only at speeds below 30 cm/s and in lower proportion than out-of-phase walk or trot. Hop was the least frequent gait, and was mainly found at the lowest speeds (5–10 cm/s) and at the transition between walk and run (60–75 cm/s), which could explain its high occurrence in several mutant mice (Kullander, 2003; Beg et al., 2007; Fawcett et al., 2007; Shi et al., 2007; Serradj and Jamon, 2009; Asante et al., 2010). Also less frequent, transverse and rotary gallops occurred between 60 and 105 cm/s. Interestingly, we found that all gaits, except lateral walk, were equally adopted by mice at 75 cm/s, thus suggesting a state of instability in the neuronal networks generating and organizing locomotor gaits at that speed. In summary, our analysis demonstrates the existence of attractor and transitional gaits occurring over a wide or discrete range of speeds, respectively.

### Outcomes of locomotor programs

We next asked whether intra-limb coordination could condition the emergence of one gait over another one. Locomotion is under temporal and spatial constraints conditioned by the step frequency and stride length of individual limbs. Under that premise, we wondered if the selection of one gait over others would provide a beneficial increase of one or more locomotor parameter(s) in the mouse's speed.

#### Step frequency

**Figure 4:**
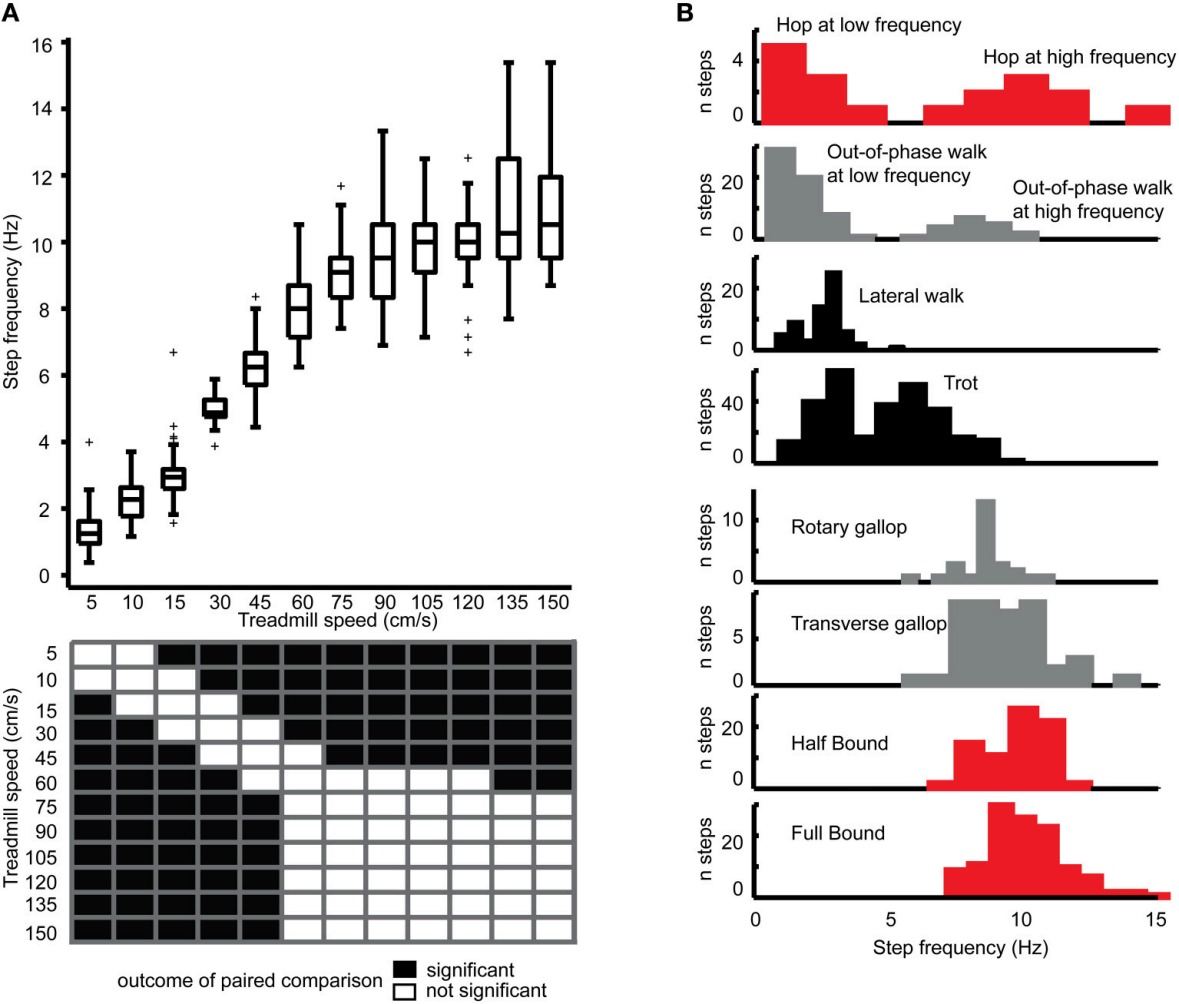
**Step frequencies in relation to speed and gait. (A)** Boxplot of step frequencies at different treadmill speeds. The upper and lower limits of the box correspond to the percentiles 75 and 25. The line within the box corresponds to percentile 50 (the median). Whiskers (vertical lines) indicate the maximal and minimal 1.5 interquartile ranges, crosses, and outliers. Outcome of statistical comparison (Kruskal-Wallis and *post-hoc* Tukey's honest HSD test) is shown in a black and white matrix at the bottom. **(B)** Color-coded histograms of step frequencies for each gait (anti-phase in black, in-phase in red, and out-of-phase in gray). Note clear bimodality for the hop and the out-of-phase walk.

We first analyzed the step frequency of the left hindlimb according to the treadmill speed (Figure 4A) and found that the step frequency increased linearly from 5 cm/s before reaching a plateau (no more significant increase) at 60–75 cm/s (*p* < 0.001, Kruskal-Wallis test and *post-ho*c Tukey's HSD test). Interestingly, as illustrated in Figure 3B, there were no predominant gaits at 60–75 cm/s, which might reflect a transient state that could preclude the neural locomotor networks from setting a particular locomotor gait. However, half-bound and full-bound emerged as dominant gaits at high treadmill speeds above 75 cm/s (Figure 3), therefore suggesting that other parameters might contribute to overcoming the temporal limitation of the step frequency beyond that speed.

Studying the step frequency as a function of locomotor gaits (Figure 4B and Table 1), we found that hop and out-of-phase walk displayed a clear bimodal distribution at low and high frequency. Although the lateral walk, trot, and half-bound also displayed an apparent bimodal distribution, this was likely due to the discrete sampling of our data (at given treadmill speeds). Therefore, except for hop and out-of-phase walk, all other gaits were treated as unimodal. Lateral walk displayed the narrowest range of step frequencies (1–4 Hz, peak at 2.6 Hz), while trot covered the widest range of step frequency (1–10 Hz with a peak below and above 5 Hz). Rotary and transverse gallops were present above 5 Hz and were similar in terms of step frequency. Half-bound and full-bound showed the highest mean step frequency.

As expected, there was an effect of gait on the step frequency (statistical comparison of distributions from Figure 3B, *p* < 0.001, Kruskal-Wallis test, paired comparison by Tukey HSD test). The step frequency during trot was significantly different from that during other gaits, with the exception of hop at low step frequency. Conversely, the step frequency during half-bound was different from that during walking gaits (out-of-phase walk and hop at low frequency, lateral walk and trot), but not different from that during half-bound, rotary gallop, and transverse gallop, as well as out-of-phase walk and hop at high frequency. Although trot provided an advantage over slow gaits with a faster step frequency, opting for full-bound over other running gaits could not be explained by an increase in step frequency.

#### Limb trajectory

**Figure 5:**
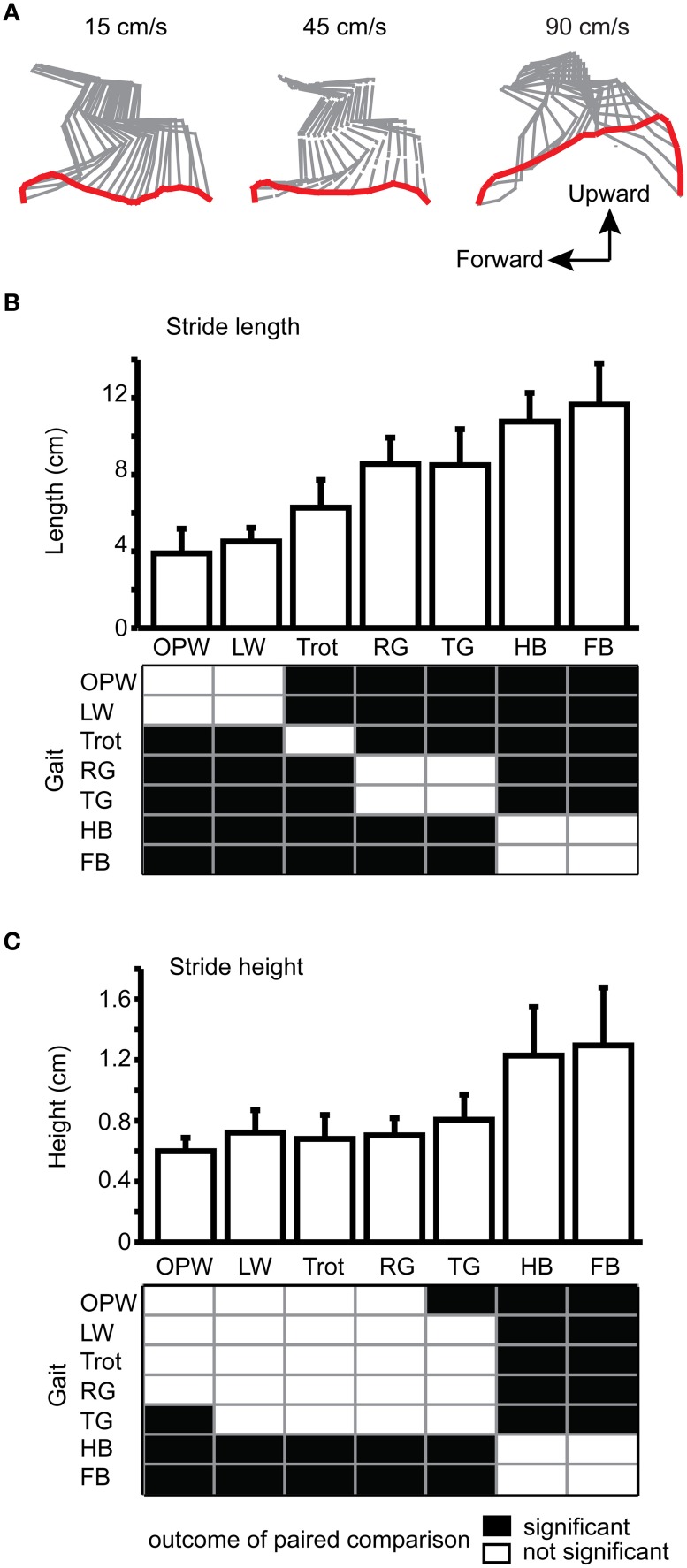
**Stride length and height for each gait. (A)** Stick diagrams of the left hindlimb during the swing phase at 15, 45, and 90 cm/s. Mean and standard deviation of **(B)** the stride length and **(C)** the stride height for each gait. Outcome of statistical comparisons (Kruskal-Wallis and *post-hoc* Tukey's HSD test) is shown in a black and white matrix at the bottom of each graph. OPW, out-of-phase walk; LW, lateral walk; RG, rotary gallop; TG, transverse gallop; HB, half-bound; FB, full-bound.

Figure 5A illustrates typical examples of hindlimb trajectories at three representative treadmill speeds: slow walking (15 cm/s), fast walking (45 cm/s), and running speed (90 cm/s). The maximal length and height of individual hindlimb strides were analyzed according to the gait (Figure 5B). There was an increase in stride length when the mouse switched from lateral or out-of-phase walk to the trot (from less than 4 cm to about 6–7 cm), and another increasep- when the animal adopted either transverse gallop or rotary gallop. The stride length reached a maximum of 11–12 cm for both half-bound and full-bound.

Because the difference between half-bound and full-bound may lie in the entire trajectory of the limb, we then quantified the maximal stride height for each gait (Figure 5C). The stride height was similar (about 0.6 cm) for the out-of-phase walk, lateral walk, trot, and rotary gallop. Similarly to the stride length, the stride height was significantly higher (twice the height) for both half-bound and full-bound than for any other gaits, but both types of bound still showed similar values. However, there were some differences in the limb trajectory of the forelimb: the stride height but not the stride length was higher during full-bound than half-bound (data not shown). The enhanced stride height of the forelimb during full-bound over half-bound might result from a reduced lateral oscillation of the scapular belt due to forelimb synchronization.

### Seeking postural stability: Distribution of supporting limbs

**Figure 6:**
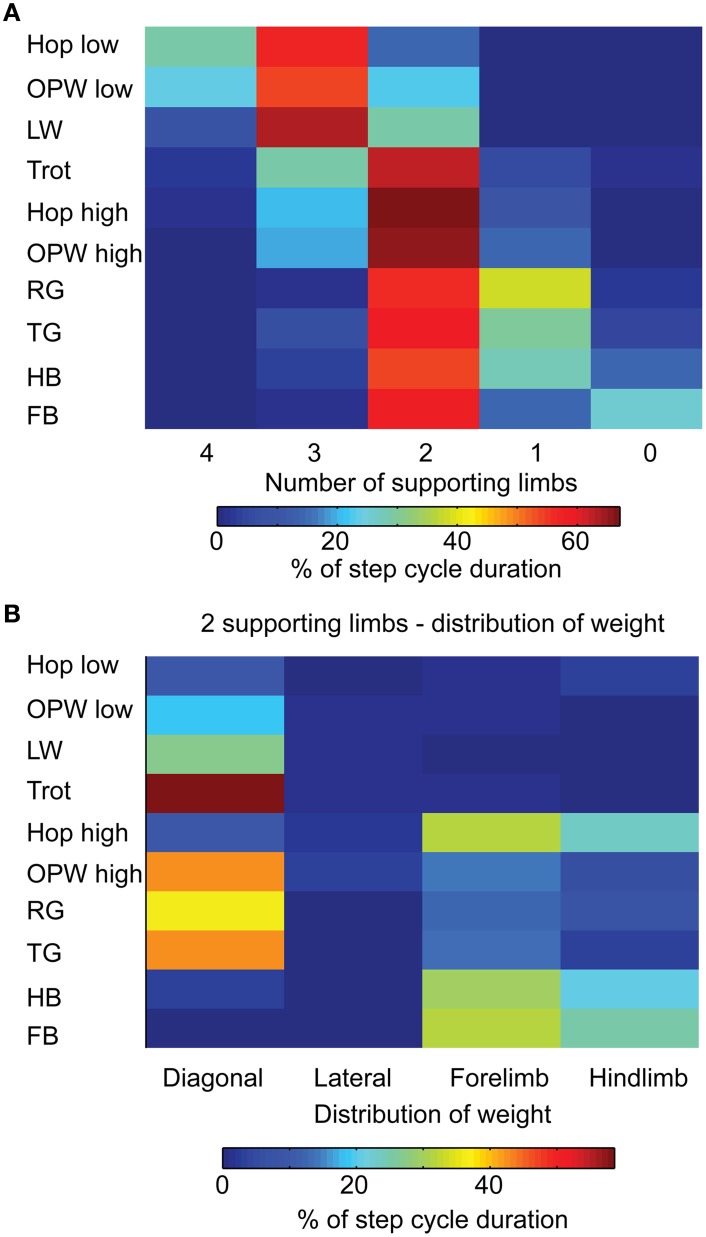
**Distribution of weight for each gait. (A)** Color-coded matrix of the percentage of the step cycle duration when mice are supported by four to no limbs (columns). Data are presented for each gait (rows). **(B)** Color-coded matrix of the percentage of the step cycle duration when mice were supported on two limbs in one of the following configurations (columns): diagonal limbs, ipsilateral limbs, forelimbs, or hindlimbs. Data are presented for each gait (rows).

Figure 6A shows the percentage of the step cycle duration spent per individual gait on a given number of limb(s). During lateral walk, hop, and out-of-phase walk at low step frequency, mice were mainly supported on three limbs. During faster gaits (trot, hop, and out-of-phase walk at high speed, gallops, and bounds), mice were supported for more than 50% of the step cycle on two limbs. Although a two-limb support was predominant during gallops and bounds, about a quarter of the step cycle was characterized by a single-limb support. This distribution was especially more frequent during rotary gallop. Because supporting the body weight on a single limb would be more hazardous for a quadruped, the larger occurrence of this support during rotary and transverse gallops might explain, in part, why these gaits were transitional rather than attractors. Similarly, a larger proportion of single-limb support during half-bound caused by an anti-phase coupling of forelimbs concomitant to an in-phase coupling of hindlimbs might explain why full-bound would emerge as an attractor gait over half-bound at the highest velocity. During a period of two-limb support, the mouse stood on the diagonal, lateral, fore-, or hindlimbs (Figure 6B). It is obvious that the impact on postural stability of these four types of support is not equivalent for a mouse. The diagonal support (characteristic of the trot) would be the most stable solution by keeping the center of mass close to the midline along the rostrocaudal axis, whereas a lateral support would be the least stable by shifting the center of mass away from the midline (typical of the pace). We indeed found that a diagonal support was the most frequent type of support and was more prominent during trot. It was also more frequent during out-of-phase walk at high step frequency, transverse gallop, and to a lesser extent during rotary gallop. As expected, mice were not found to stand on ipsilateral limbs (no pace was identified in this study), while they mainly contacted the ground with either the forelimbs or the hindlimbs during half-bound, full-bound, and hop, which is consistent with the dynamic of such gaits.

### Gait transitions

#### Gait stability and attractiveness

**Figure 7:**
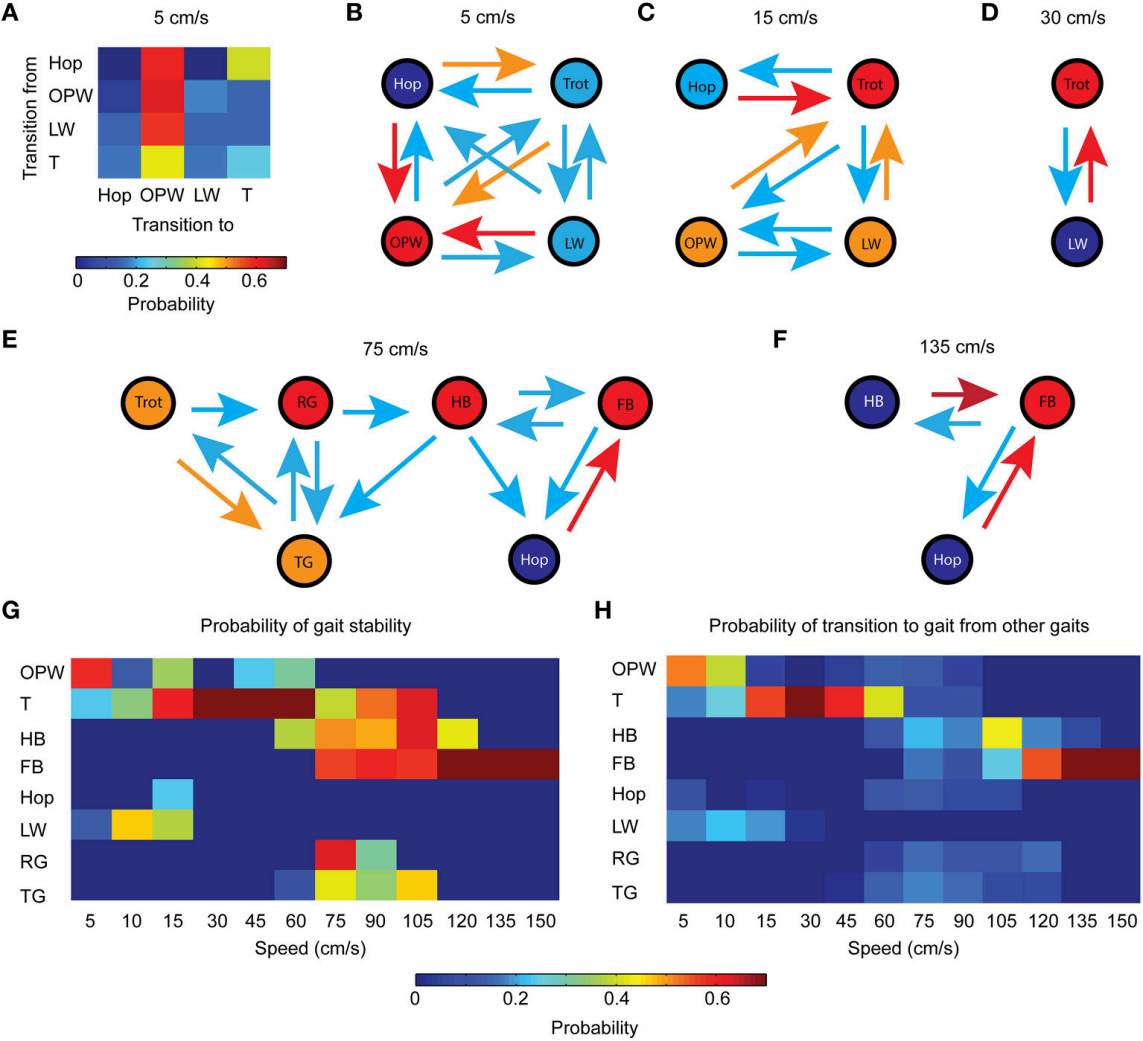
**Stability and attractiveness of gaits. (A)** Color-coded matrix of transition probability between gaits. Stability refers to a similar gait between two successive step cycles. Data correspond to a steady-state locomotion at 5 cm/s. **(B)** Graph analysis of the matrix presented in **(A)**. Gaits are represented by nodes (or vertices) and transitions by links (or edges). For sake of clarity, the diversity of color has been reduced: red = all shades of red, orange = green to orange, light blue = most shades of blue (except for deep blue, which denotes absence of link). The color of a circle indicates the stability of a gait. Similar graphs are presented at **(C)** 15 cm/s, **(D)** 30 cm/s, **(E)** 75 cm/s, and **(F)** 135 cm/s. **(G)** Color-coded matrix of the probability of stability of gaits at all investigated speeds. **(H)** Color-coded matrix of the probability that any gait will make a transition to another gait.

Based on the data presented in Figure 3, we have assumed that trot and full-bound were attractor gaits and the remainder were transitional gaits. To assess this assumption and potentially identify a directionality of transition, we represented the relationship between gaits using graph analysis. Figure 7A shows an example of the probability of transition as a color-coded probability matrix of gait transitions during locomotion at 5 cm/s. The corresponding graph is presented in Figure 7B. The probability of transition is represented as color-coded links and the stability of gait as color-coded nodes. At 5 cm/s, the most stable gait was the out-of-phase walk, thus supporting this gait is an attractor. Lateral walk and trot were much less stable than out-of-phase walk at that speed, and hop was never stabilized. Mice could break away from out-of-phase walk, but it occurred very rarely.

At 15 cm/s, trot was the most stable and considered as an attractor gait (Figure 7C). Out-of-phase and lateral walk had a preferred direction toward trot but could sometimes lead to one another. The link between out-of-phase walk and hop was broken at that speed, hop being either stable or strongly biased toward trot. At 30 cm/s (Figure 7D), trot was the only gait adopted by mice. When lateral walk appeared, it was unstable and led to trot. At 75 cm/s, there was no attractor gait, but rather a diversity of stable gaits (Figure 7E). The probability of transition was low in every direction except from the trot to the transverse gallop or from the hop to the full-bound. When leaving trot (an anti-phase gait) for in-phase gaits, rotary gallop was the only direct access to half-bound, and half-bound the only one to full-bound or hop at high step frequency. At 135 cm/s (Figure 7F), full-bound was the most stable, thus supporting this gait as an attractor gait. Hop and half-bound were unstable and invariably led to full-bound.

The stability and attractiveness of each gait across all speeds is summarized in Figures 7G,H, respectively. Except for the hop, all gaits displayed stability at least over a discrete treadmill speed (Figure 7G). As expected, trot and full-bound presented the widest range of speeds with a strong stability, demonstrating that these gaits were attractors. Out-of-phase walk and half-bound showed strong to moderate stability across a wide range of speeds, but the stability was generally less than for trot or half-bound. Lateral walk was stable at 10 and 15 cm/s, and both gallops between 75 and 105 cm/s. Regarding the attractiveness of gaits (Figure 7H), the trot and full-bound displayed the widest range of strong probability of transition, further supporting that these gaits are attractors. The other gaits were associated with weaker probability of transition, confirming their role as transitional gaits.

#### Are transitions toward gaits predictable or random?

**Figure 8:**
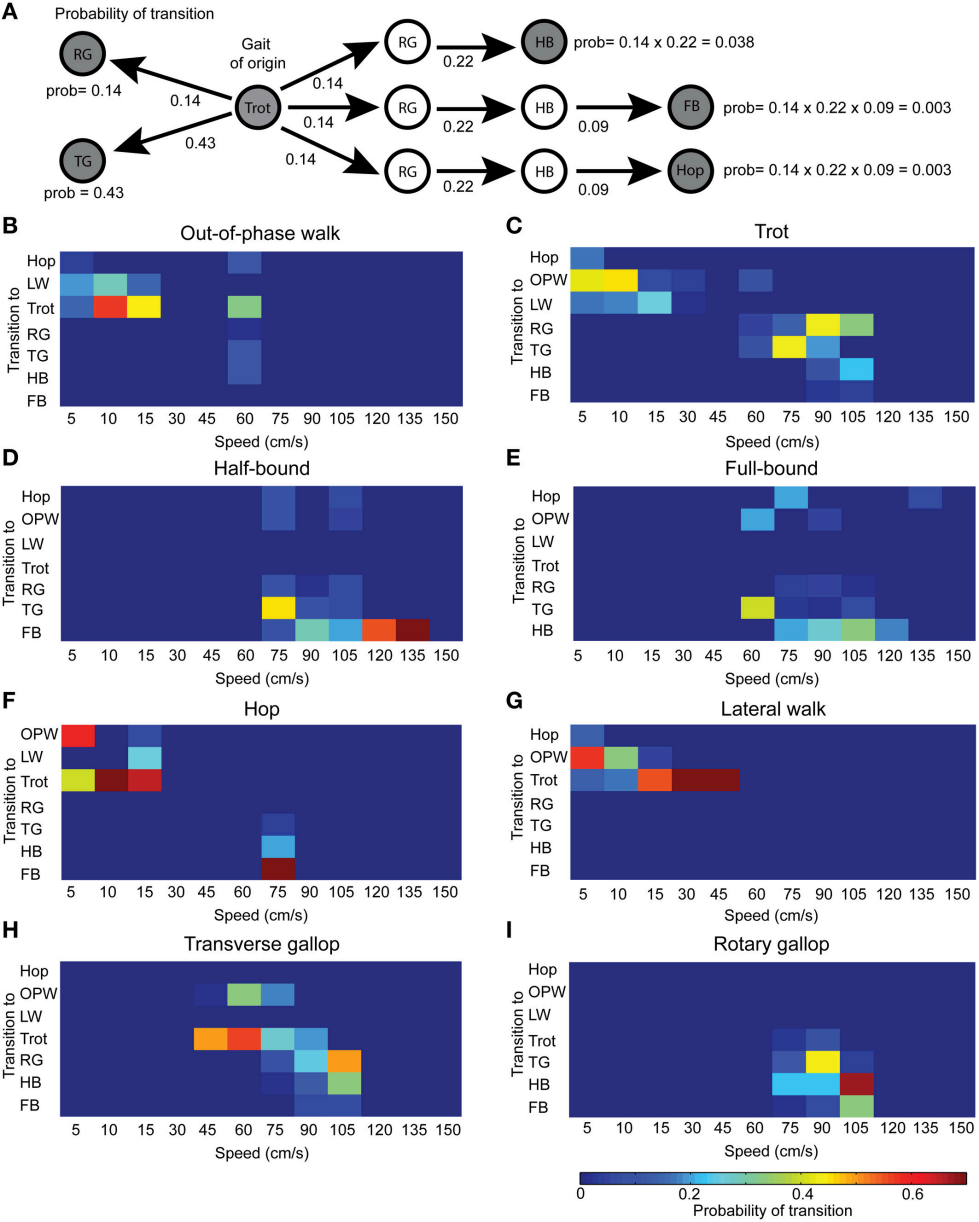
**Probability of transition for all gaits. (A)** Example of probability calculation. Probability of transition is straightforward for neighbors (path length of 1 link) but requires multiplication of probability for longer path length (up to 4 links). We used graph analysis to find the shortest path (highest probability) between two gaits. **(B–I)** Color-coded matrix of the transition probability for all speeds.

To evaluate whether transitions between gaits are predictable or occurred randomly, we analyzed the probability of transition from each gait to any other gait including those separated by 2–4 links. An example of the calculation is shown in Figure 8A. Out-of-phase walk was clearly biased toward trot, even when it reappeared at 60 cm/s, and to a lesser extent to lateral walk (Figure 8B). Although stable, mice could break away from trot and did toward out-of-phase walk at low speed and gallops at high speed (Figure 8C). As expected, half-bound was drawn toward full-bound, except at 75 cm/s where it mainly led to transverse gallop (Figure 8D). Full-bound presented the lowest values of transition probability. Transitions from full-bound mainly occurred toward half-bound, but could also lead to transverse gallop, out-of-phase walk, or hop (Figure 8E). These results suggest that out-of-phase walk is an initiation gait for locomotion and tends to lead toward walking trot. Our data also suggest that half-bound is the gateway to full-bound, which is the attractor gait at the highest running speeds.

Regarding purely transitional gaits, we found that hop was biased toward out-of-phase walk at the lowest speed and toward trot as it became the attractor (Figure 8F). We found a similar phenomenon for lateral walk (Figure 8G). Transverse gallop occurred over a wider range of speeds than rotary gallop. The transition to trot was favored at 75 cm/s and below and to rotary gallop above that speed (Figure 8H). The transition probability to half-bound was moderate at 105 cm/s. The main access to half-bound was via rotary gallop, which could also lead to full-bound. There were some transitions to transverse gallop, especially at 90 cm/s (Figure 8I). Overall, these results show that transitions do not occur in random directions but are rather biased, and thus predictable.

## Discussion

Using kinematic analysis on individual step cycles during treadmill locomotion at steady speed, we showed that the mouse displays a wide repertoire of locomotor gaits. We identified trot and full-bound as attractor gaits at walking and running speeds, respectively. Moreover, these gaits were preceded by semi-attractor gaits: out-of-phase walk and half-bound. We use the term “semi-attractor” because these gaits were more stable (several contiguous step cycle) than transitional gaits, but appeared as the attractor only at a given speed. By contrast, lateral walk, hop, and rotary and transverse gallops were less robust and less stable, emerging as transitional gaits between these attractor gaits.

### Methodological considerations

To study locomotor gaits at steady speed, we used a treadmill belt, which offers some advantages over other systems (e.g., the catwalk). Although both approaches share similar behavioral outcomes, they also exhibit contextual discrepancies, leading animals to adopt different locomotor gaits (Wetzel et al., 1975; Blaszczyk and Loeb, 1993; Herbin et al., 2007). The catwalk likely has an advantage in exploring a more natural locomotor behavior, with acceleration and deceleration phases (Bellardita and Kiehn, 2015). Nevertheless, the limited length of the catwalk (usually 1 m) limits the number of contiguous steps, in contrast to treadmill locomotion. Using the catwalk or treadmill, the challenge still remains to motivate walking or running in the mouse. Running gaits in the catwalk likely reflect a flight reaction to escape the experimenter at the gateway of the catwalk (Bellardita and Kiehn, 2015) or a noxious stimulation by pinching the tail of the mouse (Serradj and Jamon, 2009). Similarly, mice learn to walk and run on a treadmill to avoid the electrified grid or the hand of the experimenter during initial training. Although treadmill locomotion might not be less stressful during subsequent testing than the catwalk, the treadmill locomotion allows ones to study locomotor gaits over a wide range of speeds and at steady speed.

### A dynamic system with attractor, semi-attractor, and transient gaits

Locomotion is a dynamic process, which depends on intrinsic and extrinsic properties. The intrinsic properties reflect the current status and the history of the system and its sub-systems, which are embedded in the anatomy and physiology of spinal cervical and lumbar locomotor circuits, and its supraspinal descending inputs.

Using neonatal locomotor studies, mouse genetics have previously shown that manipulating genes can reorganize the spinal locomotor circuit. This neural rewiring consequently can reduce or increase the diversity of locomotor patterns, thus leading to a unique and strong left-right synchronization or an increased variability in left-right coordination (Kullander et al., 2001a,b; Beg et al., 2007; Fawcett et al., 2007; Iwasato et al., 2007; Rabe et al., 2009; Rabe Bernhardt et al., 2012).

Moreover, the neural circuit undergoes massive changes during development, thus giving rise to functional changes at the cellular, systemic, and behavioral levels. This translates into the acquisition of new locomotor gaits, as illustrated by crawling or rolling in the infant, which eventually switches to a walking then running pattern in the toddler (Forssberg, 1999; Lacquaniti et al., 2012). Similarly, gallop does not emerge prior to the 2nd postnatal week in the rat (Iwahara et al., 1991), and likely in the mouse as well. New locomotor patterns can also emerge to ensure functional compensation or recovery in patients or animal models following spinal cord injury (Barrière et al., 2008; Tester et al., 2011, 2012), neurodegenerative diseases such as Parkinson's (Morris et al., 1996, 2001; Amende et al., 2005) and Down syndrome (Parker and Bronks, 1980; Hampton et al., 2004), or even environmental manipulations (split-belt treadmill) (Thibaudier et al., 2013; Thibaudier and Frigon, 2014).

In addition, the dynamic of locomotor gaits also depends on extrinsic properties, such as the environment and the context in which the mouse evolved. Laboratory mice were fed *ad libitum* and kept in small cages are not exposed to a rich and life-threatening environment; there is hence no need to seek food and water or to escape potential predators, except occasionally the mouse's own littermates and the experimenter. In the artificial and controlled settings of our laboratory, there is therefore no reason or need for the mouse to experience and adopt a wide range of locomotor gaits, thus explaining the predominance of certain gaits at walking and running speeds in previous locomotor studies in the mouse (Herbin et al., 2004, 2006, 2007; Serradj and Jamon, 2009; Talpalar et al., 2013; Borgius et al., 2014) and in larger animals, such as the cat (Wetzel et al., 1975; Blaszczyk and Loeb, 1993; Frigon et al., 2014). Therefore, by their high probability of occurrence (Figure 3), their stability (Figure 7G), and finally their attractiveness over other gaits (Figure 7H), preferential gaits were defined as attractor gaits over other ones, which were consequently considered as transitional gaits.

#### Attractor gait: Trot and full-bound

As previously reported during over-ground and catwalk locomotion (Serradj and Jamon, 2009; Talpalar et al., 2013; Borgius et al., 2014; Bellardita and Kiehn, 2015), we identified the trot as a preferential or attractor gait during treadmill locomotion. Its large spectrum of stride frequency over a wide range of treadmill speeds allowed us to confirm its preferential use at walking and moderate running speeds. Trot was characterized by a robust alternation of hind-, fore-, and ipsilateral limbs (i.e., anti-phase coupling) and consequently a robust synchronization of diagonal fore-hindlimbs (i.e., in-phase coupling), likely resulting from a well-orchestrated and coordinated reciprocal inhibition between spinal locomotor circuits working in concert with sensory feedback and supraspinal descending control. This fore-hindlimb synchronization likely contributes to a better distribution of the mouse body weight support on its diagonal limbs during stance, thus keeping the animal's center of mass along its midline. In addition, this synchronization of diagonal limb in conjunction with a larger stride length than during out-of-phase walk and lateral walk also likely prevents ipsilateral limbs to get in the way of each other during the swing, therefore ensuring an optimal postural stability.

At the highest running speeds, we identified full-bound as an attractor gait. Surprisingly, there has been little evidence in the literature until recently that wild-type mice were capable of galloping or bounding. Indeed, previous kinematic studies showed that C57BL/6J mice tended to fast trot rather than gallop at the highest treadmill speeds (Herbin et al., 2004). Although gallops and bounds have been reported during brief acceleration phases on a treadmill (Herbin et al., 2004, 2006, 2007), in a catwalk (Bellardita and Kiehn, 2015), and in a catwalk following noxious stimulations (Serradj and Jamon, 2009), they were observed only for a few strides. Full-bound, as a high-speed running gait, is highly demanding on energy, and calls for a high motivational state (Heglund and Taylor, 1988), as suggested by the importance of the reward circuitry and especially the maturation of the nucleus accumbens and the neurotransmitter dopamine in high voluntary running rats over more sedentary rats (Garland et al., 2011; Roberts et al., 2014). Moreover, as mentioned in the previous section, the environment and the context can shape the emergence of gaits. Full-bound is necessary in a normal environment for seeking a moving prey or escaping a predator. Not surprisingly, our initial attempts to evoke gallops and bounds during locomotion at steady speed failed with 2- to 3-month-old mice (data not shown). It will therefore be important in the future to determine whether locomotor training can maintain running gaits in aging mice.

#### Semi-attractor gaits: Out-of-phase walk at low step frequency and half-bound

Semi-attractor gaits were defined as more stable than transitional gaits but over a narrower range of speeds than attractor gaits. Our analysis of locomotor gaits as a function of inter-limb coupling allowed us to identify a new gait: the out-of phase walk, which predominated over other gaits at very low speed. This gait was characterized by an out-of-phase coupling of hindlimbs, a loose anti-phase coupling of ipsilateral forelimb-hindlimb, and a relatively more robust anti-phase coupling of forelimbs. In that sense, out-of-phase walk was an attractor over a very narrow speed range and was therefore considered as a default gait emerging while the mouse was initiating locomotion, exploring its environment, or slowing down its speed. Indeed, when trot was generated at very low speed, it tended to lead back to an out-of-phase walk (Figure 8C). As such, the emergence of alternation in forelimbs, then to forelimb-hindlimb, and eventually to hindlimbs suggests that supraspinal descending inputs recruit primarily the cervical spinal locomotor circuit prior to the lumbar one, thus likely ensuring a postural stability on four limbs prior to movement initiation with forelimbs.

We also identified half-bound as a semi-attractor gait. Like out-of phase walk at slow speed, most locomotor gaits tended to lead to half-bound at low running speed over a narrow speed range, thus justifying the term of semi-attractor over attractor for half-bound. During half-bound, hindlimbs were in-phase, while forelimbs were out-of-phase. Interestingly, half-bound with its out-of-phase forelimbs appeared to emerge from rotary and transverse gallops with their anti- and out-of-phase forelimbs, but seemed to precede full-bound with its synchronized forelimbs. Therefore, there was a gradual switch from an anti-, to an out-, and then in-phase coupling of left-right forelimbs with increasing speed. Notably, this shift in the coupling of left-right forelimbs occurred at higher speeds in comparison to that of the hindlimbs. From a biomechanical viewpoint, the hindlimbs with stronger and larger extensor muscles than forelimbs are likely more efficient at propelling the animal body forward.

#### Transitional gait: Hop, lateral walk, out-of phase walk at high frequency and gallops

Hop was found at low and high step frequency. At low step frequency, hindlimb synchronization occurred rarely and always led to out-of-phase walk. It is reminiscent of the hop reported in frogs and toads at slow speed (Reilly and Jorgensen, 2011). At high step frequency, the hop resembled the jump or the leap in the frog and was intercalated with half-bound and full-bound in the mouse. Hop differed from bound by its longer duty cycle of the stance phase, thus suggesting a slight deceleration at high locomotor frequency.

Lateral walk was present up to 30 cm/s but never occurred as a dominant gait. Surprisingly, lateral walk is largely adopted by other rodents, such as the guinea pig and the rat (Hildebrand, 1976), while it was clearly less frequent in the mouse (this study). Out-of-phase walk reappeared at fast walking (and slow running) speeds and usually led to trot and rarely to gallops or bounds, suggesting it acted as a transitional gait during a decelerating phase. Gallops arose directly from trot and bridged the transition between trot and both half-bound and full-bound. The postural instability of the gallop was probably due to the larger occurrence of body weight support on a single limb, increasing the likelihood of falling, therefore requiring a rapid transition toward a locomotor gait enhancing postural stability at high running speed.

### Functional implication: What do mouse genetics reveal about gaits?

Although there is an abundant literature on genetically identified spinal interneurons important to left-right coordination, less is known about flexor-extensor alternation (Zhang et al., 2014), and even less about forelimb-hindlimb coordination. Using genetic ablation and mutant studies, 4 classes of spinal commissural interneurons: dI6, V0_*D*_, V0_*V*_, and V3, have been identified as important units to bilateral coordination, based on their transcription factor expression, their Netrin-1-DCC sensitivity, and their neurotransmitter phenotype.

Indirect evidence from Netrin-1 and DCC mutants suggests that V3 spinal interneurons are involved in hindlimb synchronization (Rabe et al., 2009; Bernhardt et al., 2012), thus likely contributing to hops, gallops, and bounds. Unfortunately, genetic silencing and c-fos studies of V3 interneurons have been performed only at walking speeds (Zhang et al., 2008; Borowska et al., 2013).

Regarding V0 spinal interneurons, genetic ablations of both V0_D_ and V0_V_ lead to a bilateral synchronization of fore- and hindlimbs (as during full-bound) at all locomotor frequencies in neonatal isolated spinal cords as well as in freely walking mice (Talpalar et al., 2013; Bellardita and Kiehn, 2015). The absence of walk, trot, and gallop in these mutant mice suggests that V0 interneurons are likely involved in these locomotor gaits. More specifically, ablation of V0_V_ interneurons abolishes trot (Bellardita and Kiehn, 2015), suggesting a role for V0_D_ in walk and gallop. Although mice lacking inhibitory V0_D_ interneurons do not survive at birth, neonatal isolated spinal cord studies revealed a gradual stabilization in left-right alternation with speed (Talpalar et al., 2013) that appears to corroborate the variability we found in the left-right hindlimb coupling of wild-type mice at locomotor frequencies ≤ 2 Hz (Figure 2A), thus suggesting that V0_D_ would initiate and stabilize left-right alternation at very slow walking speeds. Because V0_D_ cannot be specifically ablated in adult mice, it remains uncertain whether V0_D_ interneurons are necessary for gallops, and by extension, in the transition from walking to running gaits.

In absence of mice lacking dI6 interneurons or their Dmrt3 and WT1 interneuronal sub-populations, we can only speculate about their functional contribution. DI6 interneurons display an altered neuronal fate in mice lacking Dmrt3, with a decreased number of inhibitory Dmrt3 commissural interneurons at the expense of an increased number of inhibitory WT1 neurons (Andersson et al., 2012; Vallstedt and Kullander, 2013). Adult mutant mice and Icelandic horses lacking Dmrt3 alternate their left-right hindlimbs with an increased stride length and duration, resulting in a slow locomotor frequency. While pace does not appear to be part of the locomotor repertoire of C57BL/6J mice, it will be interesting to see whether pace is used by Dmrt3 mutant mice, since Icelandic horses lacking Dmrt3 do not trot or gallop but preferentially pace.

Mouse genetics studies suggest a sequential and topographical recruitment of spinal interneurons as function of the locomotor speed: from V0_D_ during walk, V0_V_ and dI6 during trot, and finally V0_*V*_, dI6, and V3 during hops, gallops, and bounds. In that regard, attractor and semi-attractor gaits would rely on the robustness of activity of these interneurons and transitional gaits would emerge when dominant activity shifts from one population to another.

### Future directions

Mouse genetics have been relying on the extensive use of neonatal decerebrated or isolated spinal cord preparations in order to record ENG activities from ventral roots during fictive locomotion. Although this approach has been very informative for studying the intrinsic and extrinsic properties of spinal interneuronal circuits, the diversity of locomotor gaits we found in the adult mouse has never been reported so far in these *in vitro* studies, thus raising some concerns about neonatal and/or isolated approaches. Unfortunately, attempts to record motor activity from adult isolated spinal cords have failed up to now (Jiang et al., 1999), presumably due to hypoxia (Wilson et al., 2003). Nevertheless, we cannot exclude that the lack of diversity might reside in the developmental stage of these isolated neonatal preparations. Indeed, gallop is not evoked prior the 2nd postnatal week in juvenile rats (Iwahara et al., 1991), and it is likely also the case in the mouse. Moreover, as shown by semi-attached or decerebrated neonatal preparations (Juvin et al., 2005, 2007, 2012), the lack of locomotor diversity in isolated neonatal mouse studies could also result from a lack of convergent inputs from supraspinal descending, cervical, as well as peripheral sensory inputs.

Still technically challenging, adult decerebrated and decerebrated-spinalized mouse preparations have allowed EMG and ENG recordings during treadmill and fictive locomotion (Meehan et al., 2012; Nakanishi and Whelan, 2012). Although there is still very little information about their gaits, it will be important in the future to study supraspinal locomotor centers important in setting these various locomotor gaits.

Alternatively to these reduced preparations, kinematic and EMG recordings in the free-walking mouse are still the best way to study spinal circuits and supraspinal descending inputs important to locomotion. Although these recordings have already been performed in the mouse at walking speed (Leblond et al., 2003; Pearson et al., 2005; Tysseling et al., 2013), little is known about their locomotor gaits. Besides, EMG implants, by preventing a normal angular excursion of locomotor movements (Pearson et al., 2005), can reduce the spectrum of locomotor gaits and speed (Lemieux et al., unpublished data). With the miniaturization of EMG implants, it will be important in the future to extend the analysis of locomotor output according to the locomotor gait at walking and running speeds.

## Conclusion

In summary, the present study shows that the adult mouse displays a wide repertoire of attractor gaits as a function of speed (from out-of-phase walk to trot, to half-bound and full-bound), but can also exhibit transitional gaits (hop, lateral walk, transverse, and rotary gallops). The choice of gait depends on locomotor outcomes: the step frequency, stride length and height, and postural stability. With advances in mouse genetics, our study highlights the importance of using more objective criteria (i.e., the interlimb coupling and the duty cycle of the stance phase) to investigate the functional contribution of genetically identified spinal, propriospinal, and supraspinal neurons to locomotor gaits over a wide range of speeds in freely walking mice.

## Author contributions

ML, NJ, and FB conceived and designed experiments. NJ, ML, MR, and SC collected, assembled, and analyzed data. ML and FB interpreted data and drafted the manuscript. ML, NJ, MR, and SC revised the manuscript. All authors approved the final manuscript and are accountable for all aspects of the work in ensuring that questions related to the accuracy or integrity of any part of the work are appropriately investigated and resolved.

## Funding

This work was supported by Canadian Institutes of Health Research (CIHR; MOP-125944, SCR-132695) and International Foundation for Research in Paraplegia (IRP; P136) grants to FB. FB is supported by a Fonds de Recherche du Québec en Santé scholarship.

### Conflict of interest statement

The authors declare that the research was conducted in the absence of any commercial or financial relationships that could be construed as a potential conflict of interest.
